# Williams Pear Canning-Industrial Residues Suitable for Powdered Products: Effect of Particle Size and Acid Immersion on Physicochemical and Bioactive Properties

**DOI:** 10.3390/foods15020377

**Published:** 2026-01-21

**Authors:** Milagros Gomez Mattson, Susana Diez, Paula Sette, Rocío Corfield, Francisco Garrido Makinistian, Carolina Schebor, Lorena Franceschinis, Daniela M. Salvatori

**Affiliations:** 1 Instituto de Investigación y Desarrollo en Ingeniería de Procesos, Biotecnología y Energías Alternativas—PROBIEN (CONICET-UNCo), Universidad Nacional del Comahue, Buenos Aires 1400, Neuquén Q8300, Argentina; milagros.gomez@probien.gob.ar (M.G.M.); susana.diez@probien.gob.ar (S.D.); paula.sette@probien.gob.ar (P.S.); francisco.garrido@probien.gob.ar (F.G.M.); lorena.franceschinis@probien.gob.ar (L.F.); daniela.salvatori@probien.gob.ar (D.M.S.); 2 Instituto de Tecnología de Alimentos y Procesos Químicos—ITAPROQ (UBA-CONICET), Universidad de Buenos Aires, Av. Int. Güiraldes s/n, Ciudad Autónoma de Buenos Aires C1053ABH, Argentina; rocio.corfield@di.fcen.uba.ar

**Keywords:** pear residues, drying kinetics, powdered ingredients, gastrointestinal digestion, bioaccessible polyphenols, dietary fiber, techno-functional properties

## Abstract

Powdered fiber- and polyphenol-rich ingredients derived from pear canning residues were obtained by direct processing. Residues were subjected to acid immersion and subsequent convective drying, milling, and sieving. Drying kinetics were studied to select the best operative drying conditions (70 °C, 3 h) for both acidified (CIT) and non-acidified (C) samples. Two granulometries were also assessed (<210 and <590 μm). The resulting powders (C210, CIT210, C590, CIT590) were characterized as bioactive compounds, techno-functional fiber properties, physical and stability attributes, as well as *in vitro* bioaccessibility. All powders were rich in dietary fiber (52–54%) and exhibited a polyphenol content ranging from ~390 to 567 mg GAE/100 g on a dry basis for CIT and C powders, respectively. Also presented good hydration properties and low oil absorption. Sample C210 was particularly noteworthy due to its higher polyphenol level and better physical and stability properties. Acid immersion slightly reduced browning during drying and, although it caused a polyphenol loss (29%), CIT samples showed a better functional potential in terms of bioaccessibility of polyphenols (83 ± 6%) and of antioxidant capacity (58 ± 1%). By analyzing multiple properties, this study offers a comprehensive evaluation of simple and cost-effective biomass utilization strategies for the production of functional ingredients.

## 1. Introduction

The food-processing industry generates large volumes of by-products and residues that represent both an environmental threat and an opportunity for value generation within a circular economy framework [1]. Fruit-derived residual biomass is particularly rich in bioactive compounds, including dietary fiber, minerals, and polyphenols, which can be recovered and transformed into functional ingredients for food or nutraceutical applications [1,2,3]. However, many of the existing studies on by-products valorization focus on the extraction of a single compound type, such as pectin, specific phenolic compounds, or bioenergy precursors, but such practices often fail to recover other nutritionally valuable components of the residue [1,2]. Moreover, the integration into industrial fruit-processing operations of many of the extraction-based strategies proposed to date is still limited, mainly due to the need for complex equipment and to the generation of a residual solid that also requires proper management for disposal [2,3].

The air drying process is widely used in industry due to its operational simplicity, low cost, and flexibility in controlling drying conditions [4]. Different drying methods, including tunnel, fluidized-bed, and rotary-drum systems, have been applied to fruit wastes and have demonstrated good capacity to preserve phenolics and antioxidant activity when properly controlled [5,6,7,8]. In particular, thin-layer drying followed by grinding has emerged as a simple, scalable, and industry-compatible alternative for transforming fruit waste into stable powdered ingredients while retaining the intrinsic matrix of the raw material, without requiring prior solvent extraction [9,10]. Based on long-established unit operations in food manufacturing, this simple and cost-effective approach could be interesting for preserving waste bioactive potential while promoting the zero-waste condition [4,11].

The cultivar Williams pear (*Pyrus communis* L.), also known as Bartlett pear in North America, is widely processed for juices, cider, and canned products, each generating by-products with different structural and compositional characteristics. Compared with wastes from juice extraction, which often consist of disrupted pomace [10,12,13,14], residues arising from canning operations, particularly when mechanical peeling is applied, would retain much of the intact skin, pulp, and fiber-rich cell wall components. Research regarding the residues from mechanical peeling in pear canning remains unexplored in the current literature. Therefore, pear canning residue is a promising substrate for full valorization, as avoiding a prior extraction step would allow the retention of the entire profile of the pear’s nutritional and phytochemical constituents, including soluble and insoluble dietary fiber, minerals, and phenolic compounds. However, treatments commonly applied to plant materials prior to air drying to prevent browning (e.g., blanching, acid dips, sulphuring) may also affect their composition and structure, depending on the type and severity of the treatment [15]. To date, this aspect has received little attention in the literature on fruit waste drying.

Despite the growing number of studies exploring fruit waste powders enriched in antioxidants or polyphenols, relatively few investigate the availability of these compounds after gastrointestinal digestion, a key factor in determining their potential biological efficacy. For instance, drying conditions and particle size have shown to influence the release and bioaccessibility of phenolic compounds of different agro-industrial by-products [7,16]. Moreover, fiber techno-functional properties (e.g., hydration capacity, oil absorption), physical attributes (particle-size distribution, flowability, color), and stability-related properties are rarely evaluated in an integrated manner together with the bioactive potential, even though they are essential for predicting the performance of waste-derived powders when incorporated into food formulations [17].

The objective of this work is to analyze the direct drying–milling process of pear residues from the canning industry as an integral valorization strategy to obtain functional powdered ingredients. This study includes the residue drying behavior analysis, the assessment of compositional, physical, techno-functional properties, as well as *in vitro* bioaccessibility, as affected by granulometry and acid immersion.

## 2. Materials and Methods

### 2.1. Materials and Reagents

Williams pear residues were collected from the canning industry, CERES S.A. (Río Negro, Argentina). It is composed of seeds, cores, peduncles, peels, and pulp generated during the mechanical peeling and conditioning of pears in the process. This company cultivates its own fruits by employing certified organic farming practices, thus ensuring the absence of synthetic pesticides, herbicides, or fertilizers in the resulting fruit wastes.

For HPLC-DAD analysis: solvents for mobile phases were Milli-Q water, HPLC-grade methanol, and 85% phosphoric acid provided by Sintorgan (Sintorgan S.A., Buenos Aires, Argentina). For patulin analysis standard was purchased from Sigma-Aldrich (St. Louis, MO, USA). For polyphenol analysis, the following standards were used: (-) epicatechin and resveratrol from Santa Cruz Biotechnology Inc. (Dallas, TX, USA); ellagic acid rom LGC Standards (Teddington, UK) and gallic acid, chlorogenic acid, p-cumaric acid, ferulic acid, caffeic acid, catechin, quercetin-3-rutinoside (rutin), and arbutin from Sigma–Aldrich Chemie GmbH (Steinheim, Germany).

For *in vitro* gastrointestinal digestion, the enzymes were purchased from Sigma–Aldrich Chemie GmbH (Steinheim, Germany). To simulate the process, a solution of α-amylase (A6255, activity = 1500 U/mL) for the oral phase, a pepsin (P7000, activity = 25,000 U/mL) solution for the gastric step, and a pancreatin (P7545, activity = 800 U/mL) solution for the intestinal phase, was used.

The human colon adenocarcinoma cell line Caco-2 (ATCC), kindly provided by Dr. Guillermo H. Docena (Instituto de Estudios Inmunológicos y Fisiopatológicos, La Plata, Argentina), was used for the cytotoxicity assays.

### 2.2. Residue Safety Evaluation and Overall Characterization

The following determinations were performed on the fresh pear waste immediately after collection.

*Microbiological quality*: It was assessed by determining mesophilic aerobic bacteria, total coliforms, and molds and yeast according to the Bacteriological Analytical Manual [18]. *Escherichia coli* was evaluated following the MPN technique (ISO 16649-3:2015) [19].

*Heavy metal determination:* The heavy metal content was determined according to the AOAC Official Method 2015.01 [20], with slight modifications for inductively coupled plasma optical emission spectrometry (ICP–OES). Analysis was performed using a JY2000-2 spectrometer (Horiba, Kyoto, Japan). Calibration was performed with a multi-element standard solution containing 26 elements (100 ppm in 5% HNO_3_; MU0111, Scharlau). Data acquisition and processing were conducted using the ICP JOBIN YVON HORIBA software (version 5.4).

*Patulin determination*: Patulin content was determined according to the method described by Oteiza et al. [21]. For sample preparation, the residue was processed into juice, which was previously depectinized by treating 100 mL with 750 µL of a 20 g/L pectinase (Pectinex, Novozymes, Bagsvaerd, Denmark). The mixture was homogenized and incubated at 40 °C for 2 h in a water bath, and subsequently it was centrifuged at 120× *g* for 10 min using an equipment model Suprafuge 22 (Heraeus Sepatech, Osterode, Germany). Then, the supernatant was collected. Extraction was performed by mixing 5 mL of the supernatant with 10 mL of ethyl acetate in a shaker model SK-300 (Jeio Tech, Seoul, Republic of Korea) for 5 min. The organic layer was recovered and evaporated under vacuum at 40–45 °C. The residue was then treated with 2 mL of 15 g/L sodium carbonate. Finally, the dried extract was resuspended in 1 mL of acetate buffer (pH 4.9). Patulin was quantified using an HPLC-DAD system (Shimadzu, Kyoto, Japan) equipped with a PolyLCReliasil C-18 column (254 × 4.6 mm, 5 µm; Phenomenex, Torrance, CA, USA). The injection volume was 40 µL. The mobile phase consisted of 30 mL/L methanol at a flow rate of 1 mL/min and a column temperature of 40 °C. The limit of detection (LOD) was 20.0 µg/kg.

*Water content:* It was measured gravimetrically following the AOAC method 925.09 [20].

*pH and titratable acidity:* Determined potentiometrically according to AOAC methods 945.27 and 945.26, respectively [20].

*Lignocellulosic components:* Cellulose, lignin, and hemicellulose contents were determined according to Torres-Sciancalepore et al. [22].

*Total extractable polyphenols* (TEP): The total extractable polyphenols content was determined using the procedure described by Sette et al. [23]. Folin–Ciocalteu reagent and gallic acid as standard were used (concentration range = 0.01–0.3 mg/mL, R^2^ = 0.994). Results were expressed as mg gallic acid equivalent (GAE) per 100 g of sample.

### 2.3. Acid Immersion Pretreatment and Drying Behavior of Pear Residue

*Acid immersion pretreatment:* The fresh residue was immersed in a 0.3% citric acid solution for ~60 min, corresponding to the total time from residue collection (including transport to the laboratory) until processing. After immersion, the residues were manually centrifuged to remove excess solution and stored at −20 °C until use. These residues were designated as CIT samples, while those without prior acid immersion were considered control samples (C).

*Drying process:* Both C and CIT residues were dried by convective drying at different temperatures (50 °C, 60 °C, and 70 °C) in an oven model Venticell 111 Standard (MMM Medcenter Einrichtungen GmbH, Munich, Germany) with forced circulation (10% relative humidity, air rate of 1–1.5 m/s). Approximately 20 g of pear residue were placed in stainless-steel perforated baskets, and triplicate drying curves were obtained by weighing the samples at regular intervals until constant humidity was achieved.

*Mathematical modeling:* The resulting drying curves were mathematically described using three semi-empirical models: Page (Equation (1)), Logarithmic (Equation (2)), and Henderson and Pabis (Equation (3)). To predict the drying time (t) required to achieve a final water content of 0.2 g/g dry basis (db) at each drying temperature, the best-fitting model was applied.

*Page model*

(1) $$\frac{X}{X_{0}}=\;\exp\left(-k{\;t}^{n}\right)$$

*Logarithmic model*

(2) $$\frac{X}{X_{0}}=a\;\exp\left(-k\;t\right)+c$$

*Henderson* and *Pabis model*

(3) $$\frac{X}{X_{0}}=a\;\exp\left(-k\;t\right)$$

where X is the average water content of the sample (db) at any time; X_0_ is the initial water content (db); k is the drying rate constant; n, a, and c are empirical parameters (dimensionless); and t is the drying time.

### 2.4. Pear Residue Powders

Once the temperature and the drying time were selected, C and CIT residues were dried under these operating conditions and subsequently ground for 1 min at 1400 rpm using a hammer mill model FZ-102 (EKO, Arcano, Nanjing, China). Two granulometries were obtained by sieving the ground samples with ASTM-USA sieves: mesh 30 (particle size < 590 µm) and mesh 70 (particle size < 210 µm). Four powders were thus produced and designated as C210, C590, CIT210, and CIT590.

#### 2.4.1. Compositional Analysis of Powders

*Water content, water activity* (*a_w_*)*, pH, and acidity*: AOAC methods (Section 2.2) were followed. Water activity was determined using an Aqualab Series 3 TE (Pullman, WA, USA) hygrometer.

*Dietary fiber content:* Total dietary fiber (TDF) and insoluble dietary fiber (IDF) were measured using the enzymatic-gravimetric method with Megazyme kit (AACC 32-05, Wicklow, Ireland) [24]. Once filtered and dried, digested residues were corrected according to protein and ash content, following the AOAC standard method (991.43). Soluble dietary fiber (SDF) was calculated as the difference between total and insoluble fibers (SDF = TDF − IDF).

*Lignocellulosic components*: This was determined according to the methodology described in Section 2.2.

*Mineral content:* It was determined with an ICP–OES spectrometer, model JY2000-2 (Horiba, Kyoto, Japan) according to Diez et al. [25].

#### 2.4.2. Antioxidant Potential of Powders

For phenolic compounds and antioxidant capacity determinations, methanolic extracts were prepared according to Sette et al. [26], and measurements were performed using a spectrophotometer T60 UV-visible (PG instruments, Lecestershire, UK).

##### Phenolic Compounds

*Global composition by spectrophotometry:* The TEP of powders was determined using the procedure described in Section 2.2. In addition, non-extractable phenolic compounds (NEP), associated with cell wall components, were determined after alkaline hydrolysis to release linked phenolic groups, according to Bunzel et al. [27]. Folin–Ciocalteu reagent and gallic acid as standard were used (concentration range = 0.01–0.3 mg/mL, R^2^ = 0.994). Results were expressed as mg gallic acid equivalent (GAE) per 100 g of sample.

*Phenolic profile by qualitative*–*quantitative HPLC-DAD analysis*: Individual phenolic compounds were evaluated in methanolic extracts, following the procedure described by Gomez Mattson et al. [7]. The analysis was performed using an Agilent 1260 HPLC system (Agilent Technologies, Waldbronn, Germany) equipped with a diode array detector (DAD), controlled by Agilent OpenLab ChemStation software (version C.01.01). Separation was carried out using a Zorbax Eclipse PLUS-C18 column (150 mm × 4.6 mm, 3.5 µm particle size; Agilent Technologies) operated at 25 °C, with a flow rate of 0.5 mL/min and an injection volume of 5 µL. The mobile phase consisted of Milli-Q water (solvent A), 85% *w*/*v* phosphoric acid (solvent B), and methanol (solvent C). The linear gradient started with 99% A and 1% B, reaching 79.2% A and 0.8% B at 10 min; 59.4% A and 0.6% B at 20 min; 39.6% A and 0.4% B at 30 min; and 19.8% A and 0.2% B at 40 min. A 6 min post-run equilibrium time was allowed between injections. Detection was performed at 280 nm for arbutin, flavan-3-ols, and gallic acid; 323 nm for hydroxycinnamic acids and stilbenes; and 365 nm for flavonols and ellagic acid. Compounds were identified by comparing their retention times and UV-Vis spectra (recorded from 210 to 610 nm) with those of authentic standards. Quantification was carried out using the external standard method. Calibration curves for each standard were prepared in a range of 1–200 mg/L (R^2^ = 0.999), and the results were expressed as mg/g sample.

##### Antioxidant Capacity

Antioxidant capacity (AC) was determined by applying two different methods [25]: (1) the bleaching method of radical 2,2-azinobis-[3-ethylbenzothiazoline-6-sulfonic acid] (ABTS^+•^), calibration curve were constructed with gallic acid (concentration range = 0.003–0.05 mg/mL, R^2^ = 0.988), and (2) the ferric reducing antioxidant power (FRAP) method, with gallic acid as standard (concentration range = 0.003–0.04 mg/mL, R^2^ = 0.999). Both results were expressed as mg GAE per 100 g of sample.

##### Bioaccessibility of Powders

*In vitro* gastrointestinal digestion (GID) assays were performed on powders and water (as a control of the process) following the INFOGEST protocol [28]. A total of 1 g of the sample was resuspended in double-distilled water in a 1:4 ratio (sample:water). Oral, gastric, and small intestine phases were carried out according to the procedure described by Gomez Mattson et al. [7]. After incubation time, samples were centrifuged at 13,000× *g* for 10 min with equipment model 5804 R (Eppendorf, Hamburg, Germany), and supernatants were kept at −20 °C for further analysis. TEP and antioxidant activity by FRAP were analyzed in the supernatant using the description above (Phenolic Compounds and Antioxidant Capacity Sections). Results were expressed as mg bioactive per g of sample, and the bioaccessibility (B) was calculated according to Equation (4) [7]:

(4) $$B\;\left(\%\right)\;=\;\frac{\mathrm{mg}\;\mathrm{bioactive}\;\mathrm{in}\;\mathrm{supernatant}}{\mathrm{mg}\;\mathrm{bioactive}\;\mathrm{in}\;\mathrm{pear}\;\mathrm{waste}\;\mathrm{powder}}\;\times \;100\;$$

##### Powder Cytotoxicity

The Caco-2 cell line (ATCC) was cultured in Dulbecco’s Modified Eagle Medium (DMEM) supplemented with 10% fetal bovine serum (FBS) at 37 °C in a humidified atmosphere containing 5% CO_2_, with medium changes every 48–72 h. When cells reached approximately 80% confluence, they were detached using TrypLE diluted in PBS-EDTA (1 mM) and subcultured. For the viability assays, 2 × 10^4^ cells/well were seeded in 96-well plates and incubated for 16 days to allow differentiation into an enterocyte-like phenotype [29]. After the differentiation period, cells were exposed for 4 h to the following treatments: (1) digested water at 100%, 50%, 30%, 20%, and 10%, used as a positive control due to the known cytotoxicity of bile salts at these concentrations [30], and (2) digested powders (C210, C590, CIT210, and CIT590) at the same dilution ratios. The negative control consisted of Dulbecco’s Modified Eagle Medium (DMEM) supplemented with 10% fetal bovine serum (FBS). Following exposure, a 0.5 mg/mL solution of 3-(4,5-dimethylthiazol-2-yl)-2,5-diphenyltetrazolium bromide (MTT) was added and incubated for an additional 2 h [30]. The resulting formazan crystals were solubilized with dimethyl sulfoxide (DMSO), and absorbance was measured at 570 and 690 nm. Cell viability was calculated as the difference between Abs_570_ and Abs_690_ relative to the negative control. All conditions were tested in triplicate across two independent experiments.

#### 2.4.3. Physical Properties of Powders

##### Properties Related to Powder Stability

*Glass transition temperature (Tg):* T_g_ (onset values) of powders were determined by differential scanning calorimetry (DSC) using a calorimeter model Q200 (TA Instruments, New Castle, DE, USA) according to Sette et al. [25]. Thermograms were analyzed using Universal Analysis 2000 software v. 4.5 (TA Instruments).

*Water sorption isotherms*: The methodology described by Gomez Mattson et al. [31] was used. 0.5 g of powders were placed in open capsules and incubated at 20 °C in desiccators with different levels of relative humidity (RH) provided by different saturated salt solutions (CH_3_COONa, 22%; MgCl_2_, 33%; K_2_CO_3_, 43%; Mg(NO_3_)_2_, 52%; NaCl, 75%). After equilibrium, water content was determined according to Section 2.4.1.

*Hygroscopicity (H)*: It was expressed as g of absorbed water per 100 g of powder (db) after 22 days of incubation at 20 °C in desiccators with the NaCl saturated solution, providing 75% of RH.

##### Properties Related to Powder Flowability

Powder flowability was evaluated according to Garrido Makinistian et al. [32] methodology.

*Particle size distribution*: It was determined using a laser light diffraction equipment under the dry powder method (LA 950 V2, Horiba, Kyoto, Japan). Mass median diameter (D) was used to express average particle size, and distribution width was characterized in terms of span index.

*Angle of repose:* Repose angles within 25° and 30° represent excellent flowability, between 31° and 35° the flow is good, and between 36° and 40° the flow is fair. Values higher than 41° indicate poor flow properties.

*Bulk density, compacted density, Carr’s compressibility Index (CI), and Housner ratio:* Bulk and compacted density were informed as g/mL and used to calculate CI and Housner ratio, using Equations (5) and (6).

(5) $$\mathrm{CI}=\frac{(\mathrm{compacted}\;\mathrm{density}\;-\;\mathrm{bulk}\;\mathrm{density})}{\mathrm{compacted}\;\mathrm{density}}$$

(6) $$\mathrm{Housner}\;\mathrm{ratio}=\frac{\mathrm{compacted}\;\mathrm{density}}{\mathrm{bulk}\;\mathrm{density}}\;$$

The following scale was used to characterize flowability:
**CI****Housner Ratio****Flowability**≤101–1.11Excellent11–151.12–1.18Good16–201.19–1.25Fair21–261.26–1.34Acceptable26–311.35–1.45Poor

##### Microstructural Analysis

The microstructure of the powder particles was examined by scanning electron microscopy (SEM). The powders were mounted on an aluminum support using conductive carbon double-sided adhesive tape and then coated with gold nanoparticles using a cathodic sputter coater model 108 (Cressington Scientific Instruments, Watford, UK). The samples were examined using a scanning electron microscope Zeiss Gemini 360 (Carl Zeiss, Oberkochen, Germany), operating at an accelerating voltage of 15 kV and a magnification of 100×.

##### Superficial Color

Photocolorimetry was used to determine superficial color using a model CR400 colorimeter (Konica Minolta Co., Tokyo, Japan), following Gomez Mattson et al. [7] procedure. Glass vials containing enough powder to complete 1 cm height were used to achieve 10 measurements for each sample. Chromatic parameters were measured for illuminant C at 2° standard observers in the CIELAB color space: L* (brightness/darkness), a* (redness/greenness), and b* (yellowness/blueness).

The color change (DE2000) of CIT samples was calculated using the CIEDE2000 Color-Difference formula in comparison with C samples.

#### 2.4.4. Techno-Functional Properties of Dietary Fiber

Swelling capacity (SC), water holding capacity (WHC), water retention capacity (WRC), the retention water (RW), as well as the oil holding capacity (OHC) were determined according to Sette et al. [9]. For each property, the following equations were used:

(7) SC mLg= VhmLWdg

where V_h_ is the volume of the hydrated sample, and W_d_ is the weight of the dry sample.

(8) WHC gg=Wh − WdWd

where W_d_ is the weight of the dry sample and W_h_ is the weight of the hydrated sample.

(9) WRC gg=WP

where W (g) represents the water mass and P (g) the dry powder.

(10) $$\mathrm{RW}\;\left(\%\right)=\frac{W}{W_{t}}\;\times \;100\;$$

where RW is the percentage of retained water, W is the water mass (g), and W_t_ is the total water added (g).

(11) $$\mathrm{OHC}\;\left(g\;\mathrm{oil}/g\right)=\frac{(W_{o}-W_{d})\;}{W_{d}}$$

where W_o_ is the weight of the dried sample with absorbed oil (g), and W_d_ is the weight of the dried sample (g).

### 2.5. Statistical Analysis

Samples were analyzed in triplicate (except in color analysis), and the mean ± standard deviation (SD) is reported. Significant differences between samples were evaluated through analysis of variance (ANOVA) and Tukey’s test (α = 0.05). Drying model parameters were determined by nonlinear least-squares regression analysis. The goodness of fit was analyzed through R^2^ (determination coefficient), RMSE (root mean square error), and χ^2^ (reduced Chi-square). Infostat^©^ Software version 2016.11.17 (UNC, Cordoba, Argentina) was used for each statistical analysis.

## 3. Results

### 3.1. Pear Residue Characterization and Processing

#### 3.1.1. Analysis of Residue Reuse Viability

The initial pear residue was fully characterized in terms of its viability for use, relevant composition, and physicochemical properties (Table 1). The viability of using the residue as a raw food material was evaluated by analyzing its microbiological quality and verifying the absence of heavy metals and characteristic toxins of pears and pear-derived products, such as patulin. Acceptable hygiene indicators were observed for *E. coli* (<3 MPN/g), and the absence of patulin mycotoxin was confirmed. Mesophilic aerobic bacteria and molds/yeast levels were similar to those found by Afroz et al. [33] in pears consumed in Bangladesh (2.1–2.4 × 10^5^ CFU/g, 1.5–9 × 10^4^ CFU/g, respectively). In addition, Swailam et al. [34] reported comparable results for fresh-cut pears, with total coliforms (20–43 MPN/g) and *E. coli* (<3–9 MPN/g) using the MPN method.

A heavy metal analysis was conducted due to their potential toxicity to human health. In fruits and their residues, these elements can originate from soil and water contamination resulting from industrial activities, mining, or pesticide use. Additionally, container materials, particularly those reused for waste disposal, may introduce additional contaminants [35,36]. Among the analyzed compounds, the concentrations of As, Cr, and Ni were below the maximum limits (<0.3 mg/kg) established by the Argentine Food Code for fruits and their derivatives. Furthermore, Hg, Pb, and Cd were not detected. Together with the microbiological analysis, these results indicate that the residue meets the “viability of use” criteria established by Argentine food control authorities for its use in the development of food ingredients. It is noteworthy that current studies on the valorization of fruit residues do not include this verification, which is essential when proposing ingredients intended for food applications.

On the other hand, the viability of raw pear residue for functional ingredients development was assessed, focusing on its dietary fiber, mineral, and polyphenol content, as well as water content (Table 1). It is a high-moisture residue due to the washing step following the peeling line at the factory, and an acidity value comparable to the range reported for pear fruits (1.24–11.92 mg malic acid/g fresh fruit) by Du et al. [37].

The mineral composition, expressed in mg/100 g fresh weight, was as follows: K, 287 ± 1; Mg, 80.9 ± 0.2; Ca, 31.9 ± 0.4; Na, 6.9 ± 0.2; Se, 6.30 ± 0.03; Mn, 3.7 ± 0.8; Fe, 2.35 ± 0.01; and Zn and Cu, <0.02. These values were higher than those reported for pear residues from other geographical regions. Saquet et al. [38] found that pear skin of the Rocha variety (Portugal) contained approximately 50–70% lower concentrations of K, Ca, and Mg, fivefold lower concentrations of Fe and Mn, and threefold higher concentrations of Zn and Cu. Similarly, Vieira and Winefield [39] studied residues from European, Chinese, and Japanese cultivars, and Odagiu et al. [40] analyzed wastes from Romanian pear, reporting mineral concentrations up to tenfold lower than those observed in the present study. In all cases, K was the predominant mineral, followed by Ca and Mg, while Mn, Fe, Zn, and Cu were present at lower levels. The mineral composition of fruits depends on agricultural practices, soil characteristics, and environmental conditions. The mineralogical composition of soils from the Alto Valle region of Río Negro and Neuquén in Argentina influences nutrient availability for crops, with irrigation water contributing additional minerals and elements. Aruani and Sánchez [41] reported that the two predominant soil types (entisols and aridisols) in this region contain Fe, Cu, Mn, and Zn. Additionally, these soils contain feldspars and micas, important sources of potassium, which likely explain the high potassium content (287 mg/100 g) observed in the pear residues, making it the predominant mineral.

Regarding polyphenol content, the pear residue showed a considerable concentration (411 ± 1 mg GAE/100 g on dry basis, db), within the range reported for pear fruits by Du et al. [37] (219–668 mg GAE/100 g db, average of different varieties). In most studies, the peel and other fruit parts are separated from the pulp in the laboratory and analyzed for comparison, whereas industrial pear pomaces have been less investigated [42]. For instance, Manzoor et al. [43] reported 610 mg GAE/100 g db in peel and 345 mg GAE/100 g db in pulp from Pakistani pears, while Piluzza et al. [44] analyzed Italian cultivars, finding 990, 2800, 3020, and 5080 mg GAE/100 g db in freeze-dried flesh, peel, core, and peduncle, respectively. Differences with the present study can be attributed not only to pear variety and cultivation conditions, but also to the fact that it involves a real industrial residue, including peel, core, peduncle, and some remaining flesh tissue, providing a total polyphenol content representative of the whole pear fruit processed for canning. Compared to other reported wastes from pome fruits, the pear residue presents an interesting phenolic level. For example, in apple pomace, Gumul et al. [45] reported 89.4 mg GAE/100 g db, while Sette et al. [26] informed 462 mg/100 g db.

The Williams pear residue exhibits a TDF of 7.25 ± 0.05% (wb), with IDF being the largest fraction, contributing approximately 5% of the residue weight, while SDF represented 2%. Additionally, a balanced insoluble fiber composition (35 ± 1% cellulose, 32 ± 2% hemicellulose, 33.3 ± 0.8% lignin) was obtained. Compared to pear pomaces from other regions (e.g., Poland, Belgium, and Spain), which show more variable insoluble polysaccharide contents [46], this balanced insoluble fiber composition is particularly beneficial, enhancing digestive health and providing potential protection against inflammatory bowel disease and colorectal cancer [47]. In other fruit-based residues (e.g., grape, date, olive), this ratio is even more unbalanced, showing a higher lignin content (30–53%), 7–18% cellulose, and 5–17% hemicellulose [3,48].

It is important to note that the nutritional composition of pear residues can vary depending on factors such as pear variety, cultivation conditions, maturation stage, and processing methods. Considering pears are already recognized as a natural source of dietary fiber (2–5%) according to Du et al. [37], the high dietary fiber content observed in the residues positions the Williams pear’s by-product as a valuable ingredient for enhancing fiber content in food products.

#### 3.1.2. Drying Kinetics and Mathematical Modeling

**Figure 1:**
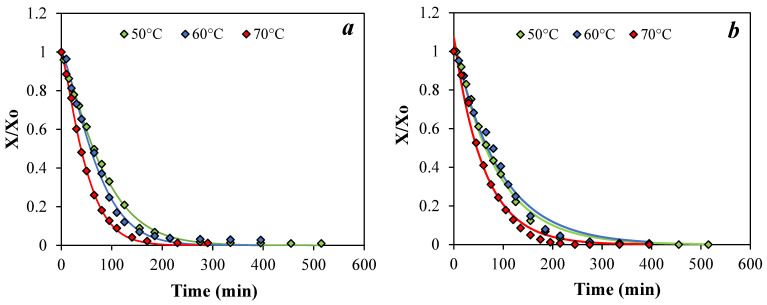
Experimental (◊) and predicted drying curves (—). (**a**) Simulation with the Page model for control samples and (**b**) with the Henderson and Pabis model (H&P) for citric acid samples, at the three temperatures studied.

Figure 1 shows the experimental drying curves obtained at different temperatures (50, 60, and 70 °C) for both C and CIT, together with those predicted with some selected mathematical models. Semi-empirical equations are widely used to describe convective drying due to their simplicity and effectiveness in characterizing the drying behavior of irregular particulate materials despite lacking theoretical foundations and being specific to experimental conditions [6]. As an example of suitable (Figure 1a) and inadequate fitting (Figure 1b), only the results obtained with Page, Henderson, and Pabis models in some specific conditions were presented. All experimental and predicted drying curves and drying rates, together with statistical parameters obtained for all predictive models (Page, Logarithmic, and Henderson and Pabis), are presented in the Supplementary Material (Figure S1, Figure S2, and Table S1, respectively).

The moisture ratio (X/X_0_) decreased continuously with drying time, and no constant-rate period was observed in any of the experiments (Figure 1). Therefore, the drying of pear residue can be considered to occur entirely in the falling-rate period, with internal moisture transport predominantly governed by diffusion mechanisms. After an initial adjustment of the samples to drying conditions, attributed to their high-water content, drying rate progressively decreased throughout the process (Figure S2), showing a drying kinetic mainly influenced by air temperature rather than by the sample type. The fluctuations observed could be ascribed to the high sample heterogeneity in terms of composition and structure of the peel, peduncle, and parenchymatic tissue that comprise it. As expected, the higher the air temperature, the higher the drying rate, with more pronounced differences observed in the C sample. The higher initial moisture content of the CIT samples, resulting from immersion in the acid solution, may account for the slower drying rate observed (Figure 1b and Figure S2(right)).

Among the tested models, the Page model provided the best fit to the experimental data, whereas the Henderson and Pabis model showed the poorest performance, showing larger deviations between predicted and experimental moisture ratios (Figure 1), consistent with the lower R^2^ values and higher error metrics (RMSE and χ^2^) presented in Table S1. The Page model was therefore used to predict the time required to reach a moisture content of 0.2 g water/g dry matter (a_w_ < 0.3). For instance, drying at 70 °C achieved the target moisture level in approximately 2.5 h for control samples, whereas acid-immersed samples required up to 3 h under the same conditions. A 10 °C increase in drying temperature reduced the total drying time by at least 1 h for both control and CIT samples. Accordingly, the drying process duration can be considered to be approximately 5, 4, and 3 h at 50, 60, and 70 °C, respectively. In this drying stage of the residues, no substantial effect of temperature was observed on the browning index (BI = 40–47) or on the total polyphenol content. The latter ranged from ≈500 to 603 mg GAE/100 g (db) in C samples and from 413 to 437 mg GAE/100 g (db) in CIT samples. Based on these results, drying at 70 °C for 3 h was selected as the best operating condition for both C and CIT samples.

Similar findings using simple semi-empirical models have been reported by other authors, who also identified the Page model as one of the most suitable for describing the drying kinetics of various food residues—such as blackberry pomace [6], tomato waste [49], and mango peel [50]—within the 50–80 °C temperature range.

### 3.2. Characterization of Powdered Products

C and CIT powders dried at 70 °C for 3 h were ground and sieved using two sieves: mesh 30 (<590 µm) and mesh 70 (<210 µm). The powders obtained (C590, C210, CIT590, and CIT210) were characterized in terms of physicochemical properties, dietary fiber and mineral content, and bioactive potential (polyphenol content and antioxidant capacity), studying the effect of particle size and acid immersion on these parameters.

CIT samples presented higher water content (CIT210 = 13.2 ± 0.3% db, CIT590 = 12.1 ± 0.2% db) compared to C samples (C210 = 10.6 ± 0.5% db, and 10.4 ± 0.5% db), whereas values for a_w_ presented similarities (between 0.186 ± 0.001 and 0.216 ± 0.001). Pear residue powders were affected by acid immersion presenting CIT samples higher acidity (2.4 ± 0.1 and 2.1 ± 0.1 g malic acid/100 g db for CIT210 and CIT590, respectively), and pH values (3.82 ± 0.02 and 3.88 ± 0.02 for CIT210 and CIT590, respectively), than C samples (1.5 ± 0.2 and 1.18 ± 0.07 mg malic acid/100 g db, pH = 4.3 ± 0.1 and 4.28 ± 0.09, for C210 and C590, respectively). These results are a consequence of the acid immersion before the drying process.

#### 3.2.1. Dietary Fiber Composition and Techno-Functional Properties

TDF, IDF, and SDF were quantified in the powdered samples, together with the structural components of the insoluble fraction and fiber techno-functional properties (Table 2). TDF contents were comparable among samples and different from those reported by Krajewska and Dziki [51] for freeze-dried powders obtained from pear residues after juice extraction. In that study, the powders exhibited higher IDF (47.2%, db) and lower SDF (10.6%, db) compared to the samples of the present study.

Particle size had a significant effect (*p* < 0.05) on TDF content, with C590 and CIT590 showing the highest values (53% and 54% db, respectively), which were 1–2% higher than those of the corresponding 210 µm samples. Regarding the distribution of soluble and insoluble fractions, a reduction in particle size resulted in a decrease in IDF. Specifically, CIT samples showed a 10% reduction in IDF (from 73.9% in CIT590 to 64.3% in CIT210, relative to TDF), while C samples exhibited a 5% reduction (from 74.7% in C590 to 70.1% in C210). In both cases, a proportional increase in SDF was observed. In general, an increased degree of particle fragmentation is associated with higher SDF content, accompanied by a reduction in the proportion of IDF [52]. A similar trend was reported by Sette et al. [9] in powders derived from maqui residues, where the higher IDF content in 590 µm powders was attributed to seed fragments retained in the coarser fractions after grinding and sieving. In the pear residue powders, fractionated through a 30-mesh sieve, the higher IDF values observed in C590 and CIT590 samples may likewise be related to the presence of seed, peduncle fragments, and stone cells characteristic of the pear’s parenchymatic tissue [53]. This effect appeared more pronounced in CIT samples, likely due to the action of citric acid, which promotes the solubilization of protopectin and soluble hemicelluloses, thereby increasing the SDF fraction [54,55].

Pear powders contain important amounts of total fiber and have a better ratio between SDF and IDF than that of other fruit residues, like, for instance, dry berry wastes, which show a high predominance of IDF [6]. SDF:IDF ratio is important for health properties and also for technological characteristics; 30–50% of SDF and 70–50% of IDF is considered a well-balanced proportion in order to obtain the physiological effects associated with both fractions [56]. For both C and CIT powders, the IDF/SDF ratio was approximately 2 in the 210 µm samples and around 3 in the 590 µm samples, indicating that all four powders have potential for use as functional food ingredients. These ratios indicate a more balanced fiber composition than the pear pomace (IDF/SDF ratio of 4) reported by Fernandes et al. [56]. This suggests the variation results from the greater structural integrity of the tissue in our residues compared to the pomace obtained from juice processing, which leads to a greater loss of soluble fiber. Moreover, regarding the composition of the insoluble fiber fraction, all samples maintained interesting levels of cellulose (36–42%), lignin (40–46%), and the insoluble fraction of hemicelluloses (28–34%), comparable to those found in the initial fresh residue. The high lignin content, arising not only from the cell walls, as in other fruits, but also from the sclereids dispersed throughout the parenchymatic tissue of pears, is particularly relevant. Lignin has been associated with the prevention of gallstone formation and the reduction in cholesterol levels [47].

Finally, the techno-functional properties of the fiber present in the studied powders were evaluated. The WHC represents the ability of the material to entrap water, while SC refers to the extent to which the matrix swells upon water absorption. The WRC corresponds to the capacity of the hydrated matrix to retain water after the application of an external force, comprising bound, hydrodynamic, and physically entrapped water [57]. As shown in Table 2, SC was not significantly influenced by particle size or acid immersion; however, powders with larger particle size exhibited higher WHC values despite having a smaller surface area per unit volume, particularly in the C samples. This behavior could be ascribed to the higher proportion of IDF of coarser powders, which may form a hydrophilic matrix in which water is entrapped [56]. Regarding water retention capacity, the most notable differences were observed between samples with and without acid immersion. CIT samples presented lower WRC values (15–17%) compared to the C samples (20–21%). Structural changes induced by immersion appeared to have a more pronounced impact on water retention capacity than variation in chemical composition (IDF/SDF ratio). Similar behavior was reported by García–Amezquita et al. [58] and Peng et al. [59] in pear peel and pomace, where the authors attributed the reduced WRC to the disruption of the insoluble polysaccharide structural network caused by acid immersion. All powders exhibited low OHC (<1.9 g/g), consistent with findings reported for pear and apple residues [59,60]. It has been suggested that the presence of lignin may contribute to oil absorption [56]; therefore, the lower IDF fraction observed in C210 samples could explain the lower OHC values obtained (Table 2). The combination of high WHC and low OHC exhibited by the powders suggests their suitability as food ingredients capable of regulating water interactions within complex food formulations and contributing to emulsifying functionality in lipid-containing matrices [52].

#### 3.2.2. Polyphenol Content and Antioxidant Capacity

**Figure 2:**
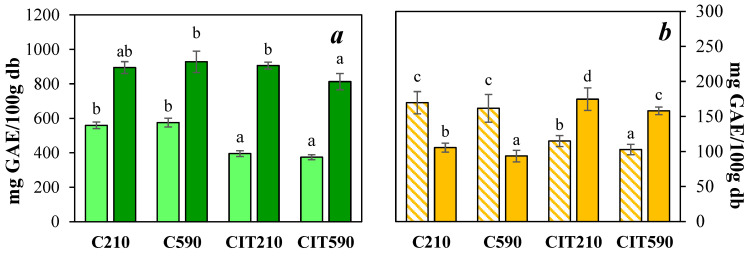
Bioactive potential of pear residue powders. (**a**) TEP (light green bars) and NEP (dark green bars), and (**b**) AC by ABTS^+•^ (yellow striped bars) and FRAP (yellow full bars) assays. Different letters indicate significant differences (*p* < 0.05) between samples.

Figure 2a presents the results for TEP and NEP, which are associated with the cell wall. Notably, NEP is much higher than TEP, particularly in acidified samples, where it is nearly twice. This finding suggests that NEP comprises a substantial fraction of the total polyphenols, which is generally not considered in studies focused on the development of plant-derived ingredients. TEP values were higher in the C samples compared with the CIT samples.

However, NEP values were similar between the C210 and CIT210 samples, while CIT590 treatment exhibited only a slight difference. Nuñez Gomez et al. [61] reported that low molecular weight polyphenols are associated with the food matrix through weak interactions, such as Van der Waals forces, hydrophobic interactions, and hydrogen bonds. When exposed to a weak acidic solvent, these interactions can be disrupted, facilitating the release and extraction of a fraction of these polyphenols. This phenomenon may partly explain the differences observed in TEP between the C samples and CIT samples, where low-molecular-weight polyphenols may be liberated into the immersion solution. On the other hand, differences in NEP were less pronounced because after alkaline and acid extraction, only those polyphenols associated with the cell wall matrix would be quantified, and not the soluble polyphenols that would be affected by such extreme extraction conditions. NEP includes low-molecular-weight compounds, such as phenolic acids, and high-molecular-weight polymeric compounds, such as proanthocyanidins and hydrolysable tannins [56]. Once synthesized, these polyphenols are transported to the cell wall, where they conjugate with macromolecules such as cellulose and proteins through ester and glycosidic linkages [61]. As a result, they may become entrapped within the fiber matrix of the powders, hindering their quantification by the regular extraction method used for TEP. Nevertheless, it is important to note that these NEPs play a key role in the large intestine: once they reach this region after passing through the gastrointestinal tract, they can be metabolized by the gut microbiota, generating compounds with potential health benefits [56,62].

Regarding AC assessed by ABTS^+•^ and FRAP (Figure 2b), the ABTS^+•^ assay, which involves both proton and electron transfer, showed higher values in the C powders, in accordance with the TEP results (Figure 2a). In contrast, the FRAP assay, based exclusively on electron transfer, yielded higher values in the CIT powders. This behavior could be explained considering that citric acid shows antioxidant activity, which, according to Švestková et al. (2024) [63], contributes to the FRAP value.

To further interpret these findings, the major individual polyphenols present in the samples were also quantified (Table 3). The results indicate that phenolic acids represent the predominant fraction, with ellagic acid being the most abundant compound. Flavonols ranked second, with quercetin as the main representative, followed by arbutin (hydroquinone β-D-glucopyranoside), and flavan-3-ols were present only as catechin. No effect attributable to particle size was observed on the distribution of these compounds. In the CIT samples, a decrease in the proportion of all phenolic groups was observed, except for arbutin. The review carried out by Saaedi et al. [64] shows that arbutin could be beneficial in the treatment of several diseases, such as hyperpigmentation disorders, cancer, central nervous system disorders, osteoporosis, and diabetes, among others.

As mentioned earlier, the initial immersion of the residue led to the leaching of polyphenols such as phenolic acids, flavonols, and flavan-3-ols (Table 3), which are more readily extracted under acidic conditions. However, the pH reduction achieved resulted in a greater retention of arbutin after drying, likely due to the inhibition of enzymatic reactions during the process.

#### 3.2.3. Bioaccessibility and Cytotoxicity After *In Vitro* Gastrointestinal Digestion

For the design and development of potentially functional ingredients derived from non-conventional sources, such as pear by-products, it is essential to evaluate not only their physicochemical characteristics but also their potential biological effects. In the case of bioactive compounds, their effectiveness largely depends on their bioavailability throughout the digestive tract and systemic circulation (Shahidi and Peng, 2018) [65]. It is also necessary to ensure that the ingredient does not exert toxic effects once consumed. An *in vitro* approach to this assessment involves evaluating both the potentially bioactive compounds and the cytotoxicity of the products obtained after *in vitro* digestion.

**Figure 3:**
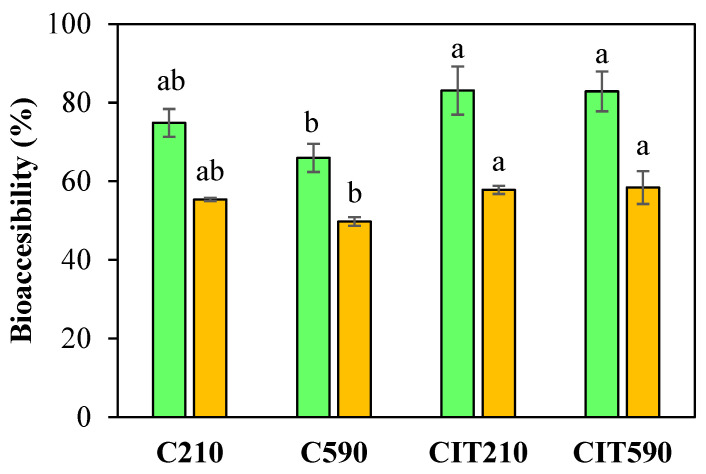
Bioaccessibility for TEP (green bars) and FRAP assay (yellow bars), analyzed after *in vitro* digestion of pear residue powders. Different letters indicate significant differences (*p* < 0.05) between samples.

Figure 3 shows the results of metabolite bioaccessibility for the pear powders, expressed as TEP and FRAP. The results indicate high B in all cases: 65–83% for TEP and 49–58% for FRAP. No significant differences were observed among samples of the same type with different particle sizes. However, powders CIT exhibited higher B.

**Figure 4:**
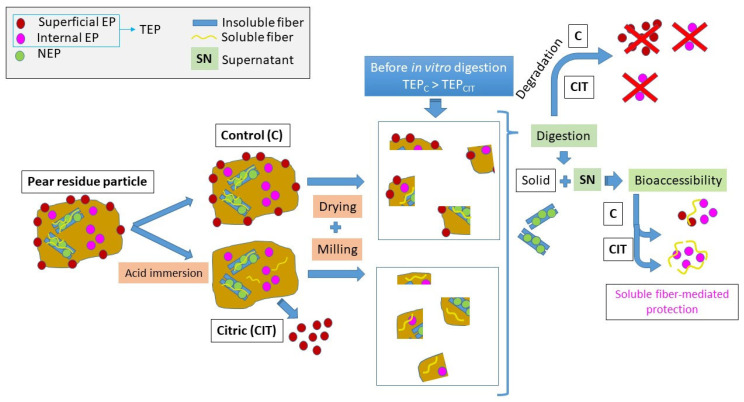
Schematic representation of the effects of processing (acid immersion–drying–milling) on the distribution and behavior of polyphenols during simulated gastrointestinal digestion *in vitro* of powdered pear residues.

This behavior is associated with the initial concentration of phenolic metabolites and antioxidant compounds present in each sample. As previously discussed, citric acid treatment may enhance the extraction of low molecular weight polyphenols by leaching, as could be observed in TEP values (Figure 2) as well as individual polyphenols determined by HPLC (Table 3). A schematic representation of polyphenol changes from initial processing to simulated digestion is shown in Figure 4. The control samples, those not treated with citric acid, despite showing higher initial TEP and FRAP values, part of these compounds correspond to extractable metabolites located in both the surface and inner layers of the powder particles that are susceptible to degradation during digestion. When CIT particles are subjected to the digestive process, the higher SDF content present in these samples could be acting as a protective matrix in the digestion medium. The SDF–TEP interaction forms a barrier-like structure that shields phenolic compounds, enhancing their stability and delivery to the intestinal lumen. This explains the lower metabolite loss in the aqueous digested phase and, consequently, the higher B, despite the diminished initial TEP and FRAP values compared to the control powders.

**Figure 5:**
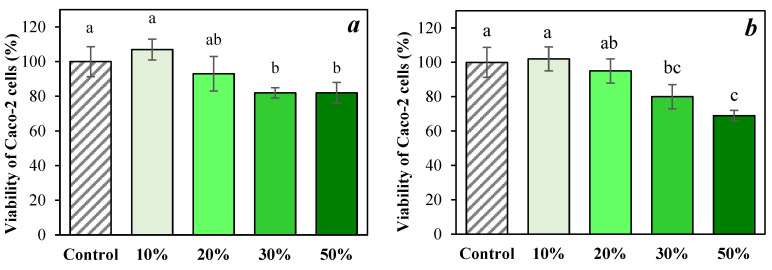

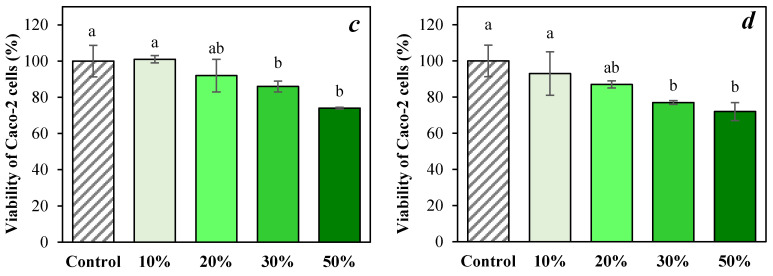
Effect of different treatments on the viability of differentiated Caco-2 cells in digested powders at different concentrations: (**a**) C210, (**b**) C590, (**c**) CIT210, (**d**) CIT590. Different letters indicate significant differences (*p* < 0.05).

Regarding the potential toxic effects of the pear by-product powders, Figure 5 shows the cytotoxicity assessed on differentiated Caco-2 cells (enterocytes). In previous studies evaluating the cytotoxicity of digested samples in undifferentiated Caco-2 cells, it has been shown that bile salts (introduced as part of the *in vitro* digestion protocol) exhibit high cytotoxicity when in direct contact with this epithelial cell line [7,30]. Consistent with these findings, the digested pear powders also demonstrated cytotoxic effects in differentiated Caco-2 cells, requiring dilution to mitigate the impact of bile salts (Figure S4). This toxicity became clearly evident at concentrations of 20% (*v*/*v*) and above.

When comparing the cell viability observed for all powder samples with that obtained after exposing Caco-2 cells to digested water (sample-free control), the digested powders yielded significantly higher viability, suggesting a potential protective effect on the cells. Similar outcomes have been reported for digested elderberry by-product powders [7] and dehydrated fruit snacks [30]. It is important to note that bile salts do not exert toxic effects under physiological conditions in humans, as there are mechanisms that ensure their efficient reabsorption [66].

#### 3.2.4. Physical Properties of Powder Products

##### Stability Evaluation

In order to estimate the physical stability of the obtained powders, several properties were evaluated: glass transition temperature (T_g_), H, and water sorption isotherms. The four powders presented T_g_ values between 45 and 51 °C, which indicated that all ingredients remained in the glassy state after drying. Several dehydrated fruit residues exhibited T_g_ in the range of 40–65 °C [25,67]. The differences observed among residues may be attributed to multiple factors, including the drying conditions (which can modify the microstructure of the material), the residual moisture content (which lowers T_g_ by plasticizing effect), and the intrinsic composition of the waste matrix. In particular, T_g_ is strongly influenced by the relative proportions of low-molecular-weight constituents (e.g., sugars, organic acids) that decrease T_g_, and macromolecular components (e.g., starch) that can increase the T_g_ of the system [68].

Sample C210 exhibited the highest T_g_ value, followed by C590, whereas the powders immersed with citric acid showed lower values (Table 4). This behavior was expected as CIT powders showed higher water contents than C powders (Section 3.2), as well as the incorporation of citric acid, which may cause the reduction in T_g_ values [69].

Additionally, water sorption isotherms at 20 °C, and the decrease in T_g_ due to the plasticizing effect of absorbed water were studied (Figure S3). Analyzing the water sorption behavior (Figure S3a), two effects could be identified, with the first and most relevant being the presence of citric acid. This acid provided a higher number of polar groups (-OH; -COOH) that promoted increased interactions with ambient moisture [70], causing higher values of water uptake when compared to control samples. The second effect was the particle size. The powders bearing the smaller particle size exhibited higher water uptake compared to their counterparts with a bigger particle size. This behavior was expected, since smaller particles present a larger surface area, increasing water adsorption [71]. In this regard, H results (Table 4) were in accordance with the water sorption results present in Figure S3a. Also, all ingredients were slightly hygroscopic (<40% *w*/*w*) [72].

Figure S3b shows the effect of water content on the T_g_ of the different powders, in a range from 11 to 75% RH. As expected, T_g_ values decreased with the increase in water content. This behavior was also observed by Ferrari et al. [67] in mango peel powders dried by hot air drying at 65 °C. CIT powders exhibited lower T_g_ values when compared to control ones. In this case, the higher H of CIT powders compared to C samples was particularly evident by the much lower T_g_ values observed at the higher RHs studied (52 and 75%).

Overall, these results showed that the effect of particle size was not so relevant regarding the water sorption and glass transition behavior. Although all the powders evaluated remained in the glassy state over the relative humidity range of 11–75%, the CIT powders presented greater limitations than control samples in terms of stability, given their higher hygroscopicity and lower T_g_ values. In particular, at RH levels that may be encountered under open packaging conditions (52–75% RH), the T_g_ of CIT powders may approach room temperature, potentially compromising their physical stability. Specifically, C210 powder proved to be the most robust and versatile formulation.

##### Superficial Color and Flowability Properties

**Figure 6:**
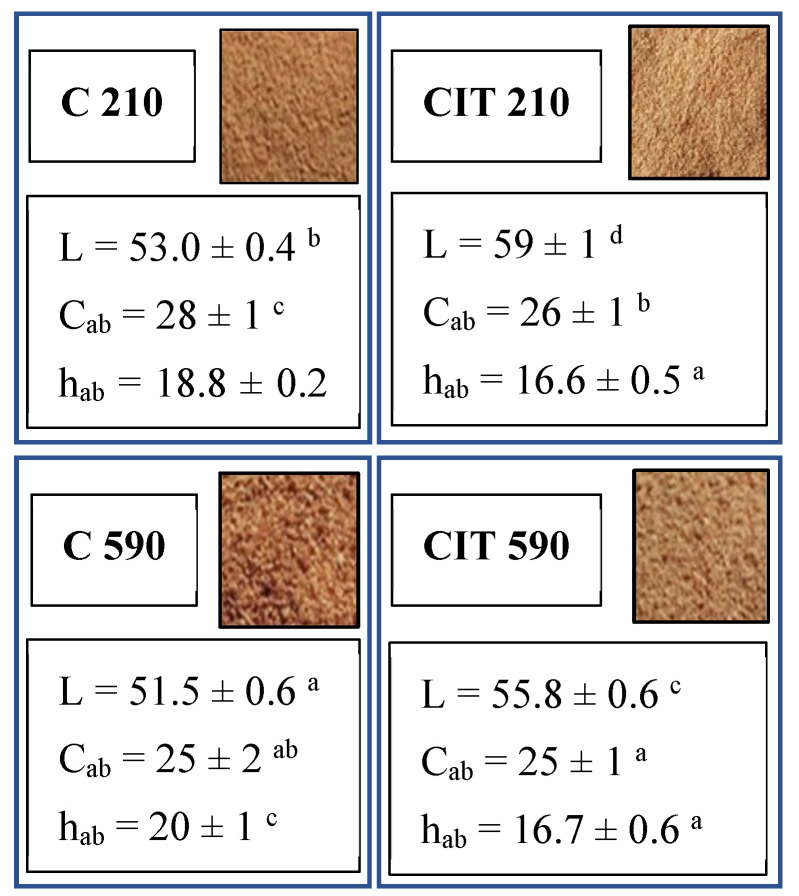
Powder images and chromatic parameters were determined for each pear waste powder. Different letters indicate significant differences (*p* < 0.05).

Figure 6 presents the images of pear waste powder ingredients together with their superficial color parameters. The typical color of dehydrated pear powders fell within the yellow–ochre to light yellow–brown hues (L = 51–59, a* = 7.4–9.3, and b* = 22.8–25.9), depending on the extent of browning and the final granulometry. Krajewska et al. [10] determined CIELab parameters for pear pomace powders, finding similar L (58–62), a* (8.82–9.63), and b* (21.20–22.2) values, after contact-drying at 60 and 80 °C.

Particle size affected luminosity, where samples 210 presented higher values compared to 590 powders, probably caused by the differences in light absorption and reflection [7]. Regarding hue and color saturation, slight differences were observed between powders, especially for h_ab_. Acid immersion yielded powders with light-yellow hues, whereas those without immersion exhibited yellow–ochre tones with slight browning (higher h_ab_). The global DE2000 values between acidified and control samples (5.3–5.7) showed a difference perceptible by the human eye [73]. Color properties of dried products are generally affected by enzymatic browning and non-enzymatic browning, such as Maillard reaction, caramelization, or polyphenol oxidation. The applied acid citric immersion may prevent some of these reactions [74]; this can be appreciated in the chromatic properties of CIT samples shown in Figure 6.

Regarding particle size distribution (Table 4), it is noteworthy that CIT samples exhibited a broader distribution (higher span values ranging from 1.89 to 2.72) compared to the C samples (ranging from 1.39 to 1.68) for both granulometries. This result could be associated with a greater tendency toward agglomeration in the CIT samples, since the formation of agglomerates promotes the generation of larger particle fractions (reflected in D_10_, D_50_, and D_90_ values for CIT samples), thereby broadening the particle size distribution. This tendency between CIT and C samples is consistent with the H results previously discussed.

**Figure 7:**
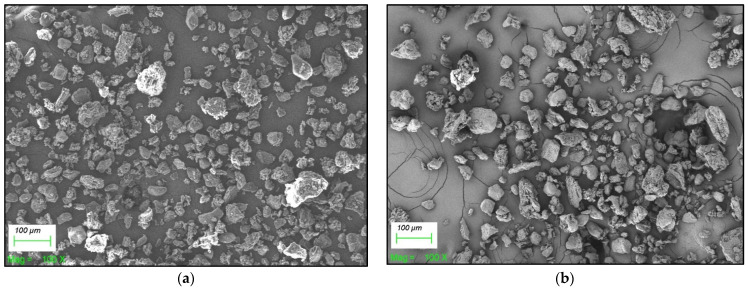
SEM micrographs of pear powders: (**a**) C210, (**b**) CIT210 at 100× magnification.

On the other hand, all four samples exhibited good flow properties. With respect to the angle of repose, the results were within the range of excellent flowability (Table 4). Similarly, the CI (11–13) and Housner ratio (1.12–1.15) values fell within the range of good flowability, except for the CIT210 sample, which showed values corresponding to acceptable flowability (CI = 20.8; Housner ratio = 1.26). This difference could be attributed to the combination of the characteristics observed in CIT210: its broad particle size distribution and the fact that it was the most hygroscopic powder. These factors promote agglomeration and increase cohesiveness, thereby obstructing flowability [75]. Differences in particle morphology can be observed in Figure 7, which shows SEM micrographs of selected powders: C210 (a) and CIT210 (b). The CIT powder (Figure 7b) showed larger particles and greater heterogeneity in diameters compared to the C powder (Figure 7a), in agreement with the higher span value observed. In addition, a certain degree of agglomeration of smaller particles onto larger ones can be observed in the CIT powder (Figure 7).

Results observed in CIT powders are consistent with previous reports on fruit powders relating acidification and higher H to enhanced agglomeration and particle size distribution broadening [76,77,78], whereas studies using hot air drying (temperatures ranging between 50 and 80 °C) indicate that residual moisture modulate the microstructure and fracture behavior during milling, which in consequence influences D_10_–D_90_ and span values [4,10].

## 4. Discussion

The valorization of a high-quality industrial Williams pear residue was achieved, integrating studies on raw material safety, drying kinetics, bioactive potential, physicochemical, and functional properties.

The Page model was suitable for describing the drying kinetics of pear residues with and without acid treatment and represents a useful tool for predicting drying time. All powders produced were rich in dietary fiber and exhibited considerable concentrations of polyphenols. They also showed a balanced profile in terms of fiber type and composition, which makes them highly suitable with respect to their technological properties and potential as functional ingredients. They can be incorporated as sources of both soluble and insoluble fiber in powdered mixes for shakes or nutritional supplements. They may also be added to breakfast cereals or instant preparations. In addition, these ingredients can be used in the formulation of cereal bars or healthy snacks, low in calories and nutrient-rich.

The powders showed good hydration properties. Those with higher WHC and WRC (coarser powders without acid immersion) can be applied to improve the nutritional profile of premixes for bakery products such as breads, cookies, and other baked goods, contributing to enhanced texture in the final product. The low OHC of the powders also suggests potential for use in fried products, since reduced oil uptake during frying generally results in lower-calorie foods. In baked products, where frying is not involved, low oil absorption may similarly be advantageous by contributing to a lighter and less greasy texture, which is particularly desirable in products such as, for instance, cookies.

However, beyond the technological applications and bioactive potential, it is highly relevant to consider the properties associated with post-production handling. In this regard, and taking all the analyzed properties into account, the powders with smaller particle size—and particularly the non-acidified ones—exhibited greater robustness in terms of properties related to physical stability.

The *in vitro* digestion studies showed a high proportion of antioxidant compounds capable of withstanding gastrointestinal conditions and remaining available for absorption, particularly in acidified powders. These results underscore the relevance of acidification as a pretreatment for pear residues, both to reduce browning during drying and also to improve the bioaccessibility of specific foods formulated with these powders. However, further studies are needed to achieve greater polyphenol retention in acidified samples.

Finally, the cell-viability assays supported the potential application of these ingredients in the development of functional foods, indicating no significant risk of cytotoxicity in *in vitro* intestinal models.

From an industrial perspective, several challenges must be considered, including the energy cost of the process. In this regard, avoiding an acid-immersion step before drying would represent the most cost-effective production strategy. Among the powders evaluated, the fine-particle control powder stands out as a practical alternative for industrial use, as it exhibited superior physical and stability properties, provides a high level of dietary fiber with a balanced composition, and showed substantial functional potential in terms of polyphenol content and bioaccessible antioxidant capacity. However, for nutraceutical applications that require differentiated levels of overall functional potential, the acidified powders would be suitable.

## 5. Conclusions

This study successfully achieved the comprehensive valorization of industrial canning pear residues by developing functional powders with high fiber and polyphenol content. The powder characterization revealed a balanced fiber profile and techno-functional properties that make them versatile ingredients for different applications. Although initial residue acidification enhanced the bioaccessibility of antioxidant compounds during *in vitro* digestion, the non-acidified fine-particle powder emerged as the most cost-effective and physically stable option for large-scale industrial use. Furthermore, the absence of cytotoxicity in cell viability assays confirms the safety of these ingredients, supporting their use in the sustainable development of new functional foods and contributing to the circular economy.

## Figures and Tables

**Table 1:** Williams pear residue safety and bioactive potential. Results are expressed on a wet basis (wb).

Residue Safety	Mean ± SD
**Microbiological quality**	
Mesophilic aerobics (CFU/g)	2.2 × 10^5^ ± 6 × 10^3^
Molds and yeast (CFU/g)	1.9 × 10^4^ ± 1 × 10^3^
Total coliforms (MPN/g)	34 ± 1
Fecal coliforms (*E. coli*, MPN/g)	<3
Patulin	Absence
**Heavy metals (mg/kg)**	
As	<0.03 *
Hg	n.d.
Cr	<0.06 *
Cd	n.d.
Pb	n.d.
Ni	<0.01 *
**Overall characterization**	
Water content (g/100 g)	86.5 ± 0.3
*a_w_*	0.979 ± 0.002
pH	4.6 ± 0.1
Acidity (g malic acid/100 g)	0.16 ± 0.10
TDF (%)	7.3 ± 0.5
IDF (%)	5.41 ± 0.04
SDF (%)	1.84 ± 0.09
TEP (mg GAE/100 g)	55.7 ± 0.1

n.d.: not detected; * concentration was below the limit of quantification (LOQ) for the metal.

**Table 2:** Dietary fiber, structural components, and techno-functional properties of powdered products.

	C210	C590	CIT210	CIT590
**Dietary fiber (%, db)**				
TDF	52.78 ± 0.06 ^a^	53.0 ± 0.4 ^b^	51.93 ± 0.01 ^a^	54.0 ± 0.5 ^b^
IDF	37.0 ± 0.9 ^b^	39.6 ± 0.3 ^a^	33.4 ± 0.4 ^c^	39.9 ± 0.2 ^a^
SDF	15.78 ^b^	13.22 ^d^	18.57 ^a^	14.16 ^c^
IDF/SDF	2.3	2.8	1.8	2.8
**Insoluble fiber composition**				
Cellulose (%)	18 ± 2 ^ab^	23.8 ± 0.4 ^b^	19 ± 1 ^a^	17 ± 1 ^a^
Lignin (%)	19.2 ± 0.6 ^a^	25.8 ± 0.4 ^b^	20.8 ± 0.3 ^a^	21 ± 1 ^a^
Hemicellulose (%)	15 ± 2 ^a^	21.4 ± 0.7 ^b^	17.5 ± 0.6 ^a^	15 ± 3 ^a^
**Techno-functional properties**				
SC (mL/g)	7.8 ± 0.2 ^bc^	7.6 ± 0.1 ^ab^	8.1 ± 0.2 ^c^	7.3 ± 0.1 ^a^
WHC (g H_2_O/g wb)	14.3 ± 0.3 ^a^	16.1 ± 0.2 ^b^	13 ± 1 ^a^	14.2 ± 0.3 ^a^
WRC (g H_2_O/g wb)	10.4 ± 0.1 ^c^	8.63 ± 0.03 ^b^	6.6 ± 0.3 ^a^	7.4 ± 0.4 ^a^
RW (%)	19 ± 2 ^bc^	21.3 ± 0.2 ^c^	15.1 ± 0.7 ^a^	17.1 ± 0.5 ^ab^
OHC (g oil/g wb)	1.31 ± 0.05 ^a^	1.6 ± 0.1 ^b^	1.5 ± 0.1 ^b^	1.42 ± 0.07 ^ab^

db: dry basis; wb: wet basis; different lowercase letters in a row indicate significant differences (*p* < 0.05) between samples.

**Table 3:** Phenolic profile determined by HPLC-DAD of pear residue powders. Results are expressed as mg per 100 g of powder (mean ± SD, *n* = 2).

Phenolic Family	Polyphenol	C210	C590	CIT210	CIT590
**Phenolic glycoside**	Arbutin	54.704 ± 0.003 ^b^	48.19 ± 0.02 ^a^	65.636 ± 0.001 ^d^	58.434 ± 0.002 ^c^
**Phenolic acids**	Chlorogenic acid	20.56 ± 0.01 ^d^	18.093 ± 0.001 ^c^	13.204 ± 0.002 ^b^	11.65 ± 0.01 ^a^
	Ellagic acid	370.2 ± 0.3 ^c^	368.82 ± 0.01 ^c^	319.44 ± 0.03 ^a^	322.04 ± 0.01 ^b^
	p-coumaric acid	0.957 ± 0.001 ^c^	0.880 ± 0.002 ^c^	0.6901 ± 0.0001 ^b^	0.76 ± 0.04 ^a^
**Flavan-3-ols**	Catechin	21.41 ± 0.04 ^d^	17.880 ± 0.002 ^c^	11.55 ± 0.03 ^b^	8.364 ± 0.002 ^a^
**Flavonols**	Rutin	17.181 ± 0.006 ^c^	15.865 ± 0.003 ^b^	10.83 ± 0.04 ^a^	11.59 ± 0.01 ^a^
	Quercetin	94.664 ± 0.002 ^d^	85.614 ± 0.001 ^c^	66.20 ± 0.01 ^b^	59.88 ± 0.02 ^a^

Different lowercase letters in a row indicate significant differences (*p* < 0.05) between samples.

**Table 4:** Physical properties for powder ingredients.

Properties	C210	C590	CIT210	CIT590
H * (g water/g db)	0.308 ± 0.004 ^b^	0.2964 ± 0.0005 ^a^	0.380 ± 0.004 ^d^	0.349 ± 0.001 ^c^
T_g_ (°C)	51.2 ± 0.5 ^b^	48 ± 1 ^a^	47 ± 1 ^a^	45 ± 2 ^a^
**Flowability**				
Response angle (°)	17 ± 1 ^ab^	16.1 ± 0.7 ^a^	16.6 ± 0.7 ^a^	18.55 ± 0.03 ^b^
δ_bulk_ (g/mL)	0.446 ± 0.003 ^a^	0.444 ± 0.006 ^a^	0.50 ± 0.01 ^b^	0.52 ± 0.02 ^b^
δ_compacted_ (g/mL)	0.512 ± 0.006 ^a^	0.49 ± 0.02 ^a^	0.623 ± 0.008 ^b^	0.62 ± 0.01 ^b^
CI (%)	12.1 ± 0.5 ^a^	11.1 ± 0.4 ^a^	20.8 ± 0.5 ^b^	13 ± 2 ^a^
Housner ratio	1.14 ± 0.01 ^a^	1.12 ± 0.01 ^a^	1.26 ± 0.01 ^b^	1.15 ± 0.02 ^a^
**Particle Size**				
D_[4,3]_ (µm)	124 ± 3 ^a^	354 ± 16 ^c^	121 ± 2 ^a^	226 ± 7 ^b^
D_10_ (µm)	42 ± 2 ^c^	92 ± 4 ^d^	30 ± 1 ^a^	36.0 ± 0.5 ^b^
D_50_ (µm)	120 ± 2 ^b^	325 ± 5 ^d^	106 ± 1 ^a^	171 ± 7 ^c^
D_90_ (µm)	209 ± 10 ^a^	637 ± 47 ^d^	231 ± 5 ^b^	503 ± 16 ^c^
Span	1.392	1.677	1.896	2.731

* values correspond to 22 days of assay. Different lowercase letters in a row indicate significant differences (*p* < 0.05) between samples.

## Data Availability

The original contributions presented in this study are included in the article and Supplementary Materials. Further inquiries can be directed to the corresponding author.

## Supplementary Materials

The following supporting information can be downloaded at https://www.mdpi.com/article/10.3390/foods15020377/s1, Figure S1. Experimental and predicted drying curves, simulated with each mathematical model, Figure S2. Drying rate vs. water content during convective drying, Figure S3. (a) Water sorption isotherms and (b) glass transition temperature vs. water content at 20 °C. Figure S4. *In vitro* digestion components on the viability of Caco-2 differentiated cells. Table S1. Statistical parameters for Page, Logarithmic, and Henderson and Pabis models at the drying temperatures studied.

## Abbreviations

The following abbreviations are used in this manuscript:

C	Control residue
CIT	Residue pretreated by immersion in acid solution
C210	Control powder with particle size <210 µm
CIT210	Pretreated powder with particle size <210 µm
C590	Control powder with particle size <590 µm
CIT590	Pretreated powder with particle size <590 µm
TEP	Total extractable polyphenols
GAE	Gallic acid equivalent
a_w_	Water activity
TDF	Total dietary fiber
IDF	Insoluble dietary fiber
SDF	Soluble dietary fiber
NEP	Non-extractable phenolic compounds
AC	Antioxidant capacity
FRAP	Ferric reducing-antioxidant power
ABTS^+•^	2,2-azinobis-[3-ethylbenzothiazoline-6-sulfonic acid] radical cation
B	Bioaccessibility
T_g_	Glass transition temperature
RH	Relative humidity
H	Hygroscopicity
D	Mass median diameter
CI	Carr’s compressibility index
SEM	Scanning electron microscopy
SC	Swelling capacity
WHC	Water holding capacity
WRC	Water retention capacity
RW	Retained water
OHC	Oil holding capacity
SD	Standard deviation
