# Supplementary material for: Williams Pear Canning-Industrial Residues Suitable for Powdered Products: Effect of Particle Size and Acid Immersion on Physicochemical and Bioactive Properties
Source: Foods. 2026 Jan 21;15(2):377. doi: 10.3390/foods15020377 (PMC12841569; doi:10.3390/foods15020377)
Supplement: Supplementary file 1 [file foods-15-00377-s001.zip › foods-4082771-supplementary.pdf]

Supplementary material

Supplementary Figures

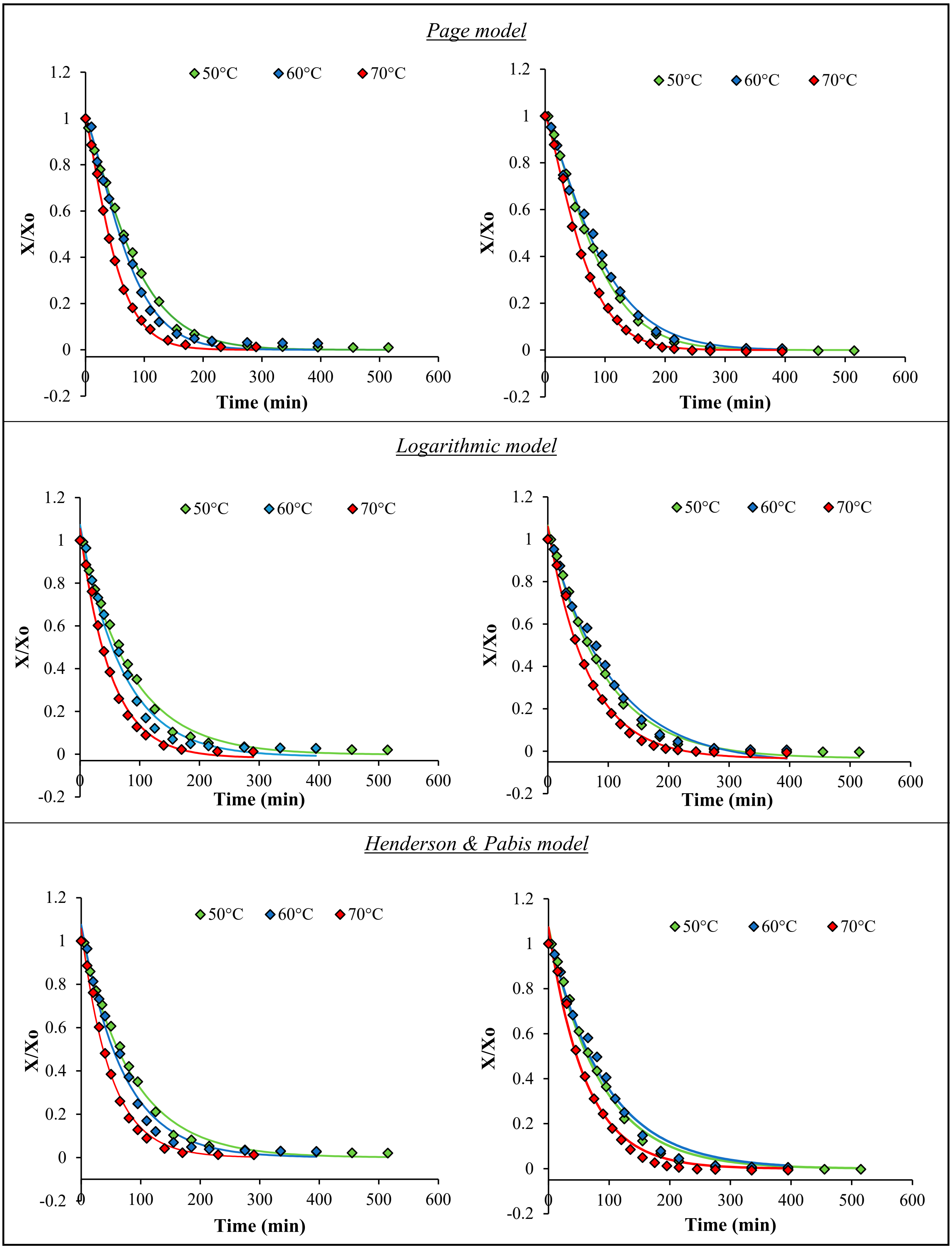

Figure S1. Experimental (◊) and predicted drying curves (—), simulated with each mathematical model, for C (left) and CIT (right) samples, at the three temperatures studied.

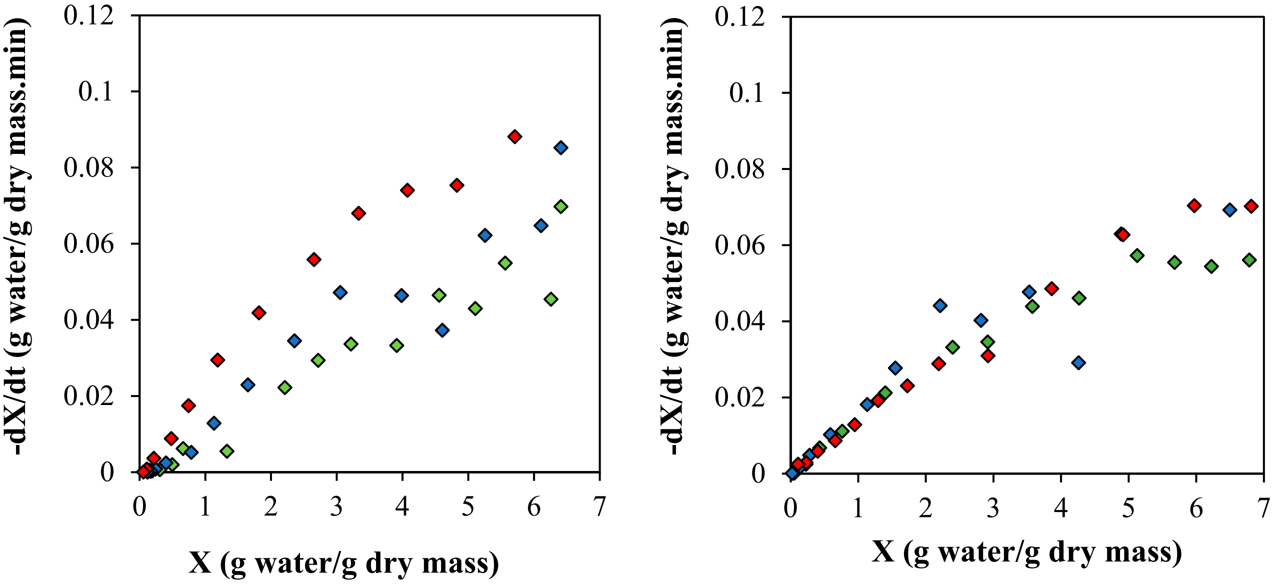

Figure S2. Drying rate vs. water content during convective drying of C (left) and CIT (right) samples at the three temperatures studied (50°C green, 60°C blue, 70°C red).

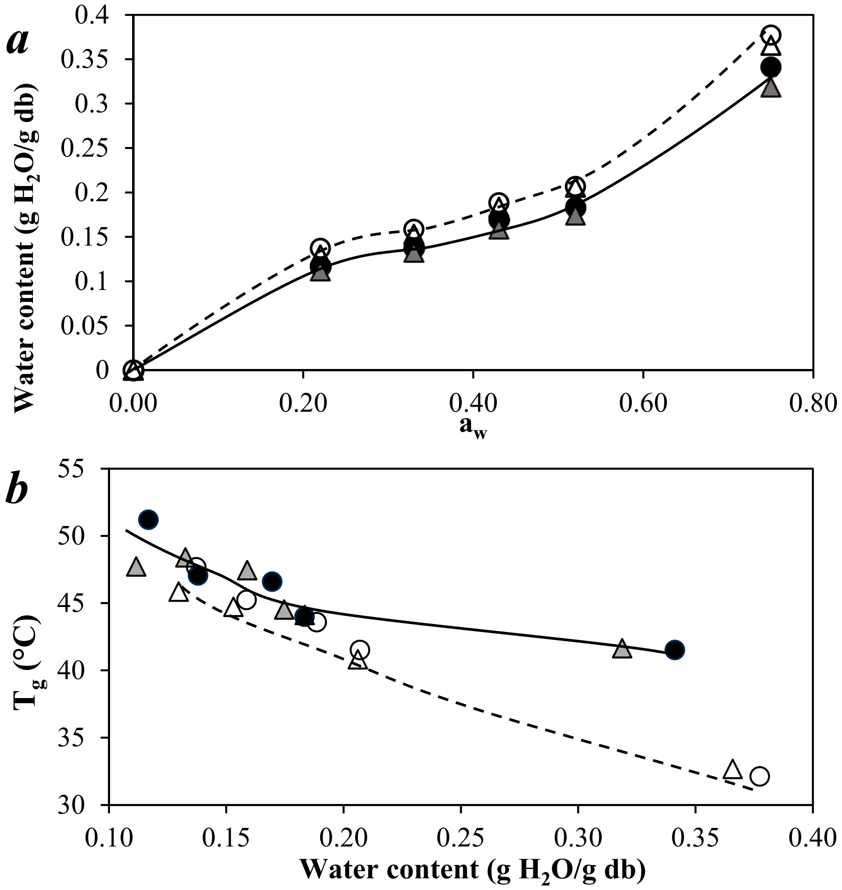

Figure S3. *a*) Water sorption isotherms and, *b*) glass transition temperature vs water content for pear powder ingredients at 20°C. Full and stripped lines represent the tendency for control and citric samples, respectively. ● C210, ▲ C590, ○ CIT210, and Δ CIT590.

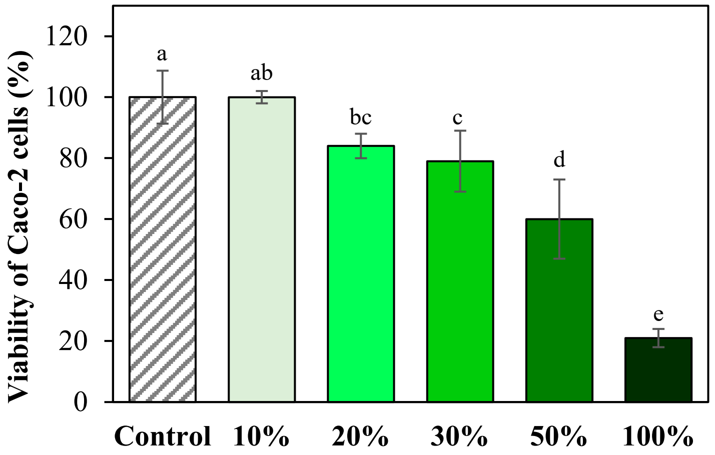

Figure S4. *In vitro* digestion components on viability of Caco-2 differentiated cells. Different letters indicate significant differences (p<0.05).

Supplementary Tables

Table S1. Statistical parameters for Page, Logarithmic, and Henderson & Pabis models for control and citric acid samples, at the drying temperatures studied.

	**Control sample (C)**		
Page model	50°C	60°C	70°C
k	0.0044 ± 0.0007 [ab]	0.00306 ± 0.00009 [a]	0.006 ± 0.002 [b]
n	1.21 ± 0.03 [a]	1.334 ± 0.003 [b]	1.31 ± 0.09 [ab]
χ^2	0.0003	0.0005	0.0003
RMSE	0.0041	0.0074	0.0038
Adj. R^2	0.998	0.996	0.998
Logarithmic model			
k	0.0117 ± 0.0001 [a]	0.0137 ± 0.0003 [ab]	0.0193 ± 0.0003 [c]
a	1.051 ± 0.008 [a]	1.085 ± 0.001 [b]	1.08 ± 0.02 [b]
c	-0.010 ± 0.009 [b]	-0.015 ± 0.004 [b]	-0.03 ± 0.02 [ab]
χ^2	0.0010	0.0019	0.0009
RMSE	0.0148	0.0249	0.1011
Adj. R^2	0.993	0.985	0.992
Henderson & Pabis			
k	0.0119 ± 0.0001 [a]	0.0142 ± 0.0002 [ab]	0.0206 ± 0.0004 [c]
a	1.043 ± 0.007 [a]	1.074 ± 0.002 [b]	1.061 ± 0.004 [b]
χ^2	0.0010	0.0019	0.0009
RMSE	0.0165	0.0259	0.0111
Adj. R^2	0.992	0.986	0.992
	**Citric sample (CIT)**		
Page model	50°C	60°C	70°C
k	0.0023 ± 0.0005 [a]	0.0027 ± 0.0006 [ab]	0.0036 ± 0.0006 [ab]
n	1.36 ± 0.05 [b]	1.29 ± 0.07 [ab]	1.34 ± 0.04 [b]
χ^2	0.0001	0.0007	0.0002
RMSE	0.0011	0.0104	0.0026
Adj. R^2	1.000	0.995	0.999

Logarithmic model			
k	0.0112 ± 0.0003 [a]	0.01 ± 0.03 [ab]	0.0149 ± 0.0002 [b]
a	1.098 ± 0.001 [b]	1.10 ± 0.03 [b]	1,103 ± 0.009 [b]
c	-0.031 ± 0.005 [ab]	-0.06 ± 0.03 [a]	-0.04 ± 0.01 [a]
χ^2	0.0010	0.0013	0.0011
RMSE	0.0150	0.0162	0.0163
Adj. R^2	0.993	0.990	0.990
Henderson & Pabis			
k	0.0120 ± 0.0002 [a]	0.0110 ± 0.0005 [a]	0.0166 ± 0.0002 [b]
a	1.074 ± 0.002 [b]	1.06 ± 0.03 [b]	1.073 ± 0.001 [b]
χ^2	0.0010	0.0017	0.0019
RMSE	0.0201	0.0233	0.0284
Adj. R^2	0.992	0.987	0.983
